# The Endoplasmic Reticulum Unfolded Protein Response in Neurodegenerative Disorders and Its Potential Therapeutic Significance

**DOI:** 10.3389/fnmol.2017.00187

**Published:** 2017-06-16

**Authors:** Paolo Remondelli, Maurizio Renna

**Affiliations:** ^1^Dipartimento di Medicina, Chirurgia e Odontoiatria “Scuola Medica Salernitana”, Università degli Studi di SalernoSalerno, Italy; ^2^Cambridge Institute for Medical Research, Department of Medical Genetics, Wellcome Trust, Addenbrooke’s Hospital, University of CambridgeCambridge, United Kingdom

**Keywords:** unfolded protein response, ER stress, protein misfolding disorders, neurodegenerative diseases, therapeutic targets

## Abstract

In eukaryotic cells, the endoplasmic reticulum (ER) is the cell compartment involved in secretory protein translocation and quality control of secretory protein folding. Different conditions can alter ER function, resulting in the accumulation of unfolded or misfolded proteins within the ER lumen. Such a condition, known as ER stress, elicits an integrated adaptive response known as the unfolded protein response (UPR) that aims to restore proteostasis within the secretory pathway. Conversely, in prolonged cell stress or insufficient adaptive response, UPR signaling causes cell death. ER dysfunctions are involved and contribute to neuronal degeneration in several human diseases, including Alzheimer, Parkinson and Huntington disease and amyotrophic lateral sclerosis. The correlations between ER stress and its signal transduction pathway known as the UPR with neuropathological changes are well established. In addition, much evidence suggests that genetic or pharmacological modulation of UPR could represent an effective strategy for minimizing the progressive neuronal loss in neurodegenerative diseases. Here, we review recent results describing the main cellular mechanisms linking ER stress and UPR to neurodegeneration. Furthermore, we provide an up-to-date panoramic view of the currently pursued strategies for ameliorating the toxic effects of protein unfolding in disease by targeting the ER UPR pathway.

## Introduction

The abnormal aggregation of misfolded proteins, which accumulate within the endoplasmic reticulum (ER) or the cytosol, characterizes several pathological conditions (Balch et al., 2008; Hartl et al., 2011).

Protein misfolding can result from genetic mutations affecting normal protein folding or derive from malfunction of the cytosolic or ER resident protein folding machinery. Many diseases result from misfolded proteins that accumulate within the ER, where they generate a stressful condition referred to as ER stress. Cells counteract ER stress by activating a group of signaling pathways termed the unfolded protein response (UPR) that coordinate a potent transcriptional program whose main purpose is restoring ER and cell function and ensuring cell survival. UPR improves folding control by increasing the expression of ER enzymes, chaperones and intracellular transporters with the aim of preventing protein aggregation and in parallel modulates the removal of misfolded proteins that would otherwise compromise cellular functions (Sitia and Braakman, 2003). Concomitantly, UPR temporarily down-regulates the protein translation rate to limit the amount of potential unfolded proteins entering the ER (Zhao and Ackerman, 2006). Moreover, UPR stimulates ER membrane synthesis and regulates the turnover of incorrectly folded proteins by fine-tuning the ER-associated protein degradation (ERAD) pathway (Meusser et al., 2005). Conversely, overtly severe or prolonged ER stress activates the UPR-dependent apoptotic cell death program (Zhang and Kaufman, 2006; Mori, 2009; Walter and Ron, 2011).

Although physically separate, there is an intimate interconnection between the protein folding machinery of the ER and cytosol. As such, increased misfolded proteins in the cytosol can compromise proteasome activity, inhibit ERAD and elicit an ER stress response, and compensatory activation of the autophagic pathway, both aimed at antagonizing the protein overload. Indeed, both proteasome activity and the autophagic pathway progressively decline in several neurodegenerative diseases resulting in proteotoxicity, chronic ER stress and ultimately lead to activation of the programmed cell death pathway (Schroder and Kaufman, 2005; Hetz et al., 2011).

Protein unfolding can be considered the initial event endorsing neurodegenerative diseases (Selkoe, 2003; Haass and Selkoe, 2007; Uversky, 2007; Makioka et al., 2010; Lindquist and Kelly, 2011; Martin et al., 2011; Roussel et al., 2013; Hetz and Mollereau, 2014; Valastyan and Lindquist, 2014) that are generally indicated as protein misfolding disorders (PMDs). Misfolded proteins are disease-specific. Among them, tau and β-amyloid (Aβ) cause Alzheimer disease (AD); α-synuclein (α-syn) mutations are a common hallmark of Parkinson disease (PD); the RNA binding proteins fused in sarcoma/translocated in sarcoma (FUS), ZNF and TAR DNA-binding protein 43 (TDP43) are associated with frontotemporal dementia (FTD) and amyotrophic lateral sclerosis (ALS); expanded polyglutamine (poly-Q) traits containing proteins cause Huntington disease (HD) and spinal cerebellar ataxias; prion proteins (PrP) are associated with Creutzfeldt-Jakob disease (CJD) (Prusiner, 1998; Knowles et al., 2014; Vidal et al., 2014).

Neuronal cells are particularly sensitive to protein misfolding in contrast to non-neuronal cells, in which cell duplications help to counteract misfolding during ER stress by repeatedly diluting unfolded peptides. By contrast, non-dividing post-mitotic neurons are completely dependent on UPR for survival. Consequently, if the misfolding is not removed and normal cell functions are not restored, UPR leads to apoptosis, leading to selective neuronal death.

In view of these observations, identifying molecules that can target UPR signaling components represents an important approach for discovering new therapies for neurodegenerative diseases. Genetic approaches such as gene therapy and RNA interference that can selectively modulate UPR in affected tissues are also under active investigation.

In this article, we review the role of UPR in the pathogenesis of neurological diseases and discuss recent progress in modulating ER stress, UPR and therefore ER homeostasis via pharmacological and gene therapy-based approaches.

## The UPR Pathways

**FIGURE 1:**
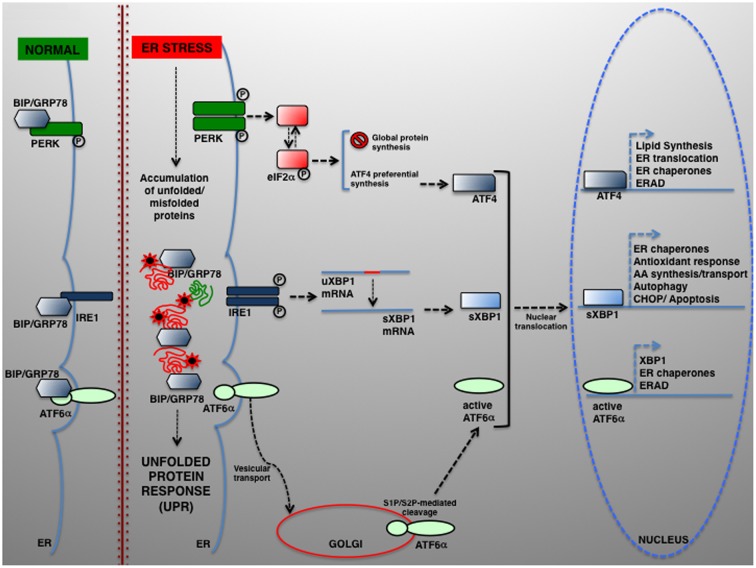
The UPR signaling pathways. In eukaryotic cells, a large portion of the total proteome is synthesized within the ER. Newly synthesized proteins can then be transported to their final destination along the secretory pathway. Several physiological and pathological conditions can hamper normal protein folding within the ER, which in turn determines the accumulation of unfolded or misfolded proteins and UPR activation. UPR is controlled by three transducers: IRE1, ATF6α, and PERK. In normal conditions, the luminal IRE1, ATF6α, and PERK regions are associated to and kept in the inactive state by the ER-resident chaperone BIP/GRP78. Upon ER stress, BIP/GRP78 dissociates and enables UPR activation. ATF6 is transported from the ER to the Golgi, where it undergoes proteolytic cleavage by S1P/S2P proteases. The cytosolic ATF6 fragment (active ATF6α) is ultimately translocated into the nucleus where it acts as a transcription factor of genes required for ERAD and modulates *XBP1* transcription). ER stress also activates PERK, which phosphorylates eIF2α to attenuate protein translation. In parallel, preferential ATF4 translation drives the expression of ER chaperones and other genes controlling autophagy, redox control and nutrient metabolism. Under severe ER stress, the PERK–ATF4 pathway controls the proapoptotic genes, including *CHOP*, which leads to programmed cell death. Once activated, IRE1 operates the alternative splicing of XBP1. The active XBP1(s) up-regulates, among others, genes encoding for ER chaperones, ERAD components and proteins involved in the lipid biosynthesis pathways.

The UPR relies on the activation of three individual modules, each represented by an ER transmembrane protein, namely PRKR-like ER kinase (PERK), inositol-requiring enzyme 1 (IRE1) and activating transcription factor 6 (ATF6) (Schroder and Kaufman, 2005). In normal conditions, PERK, IRE1, and ATF6 are kept inactive by physical interaction with the chaperone BIP/GRP78 (binding immunoglobulin protein/78-kDa glucose-regulated protein). In response to ER stress, BIP/GRP78 preferentially binds the unfolded polypeptides when they become abundant within the ER, thereby permitting PERK, IRE1, and ATF6 activation (schematically summarized in **Figure 1**).

Upon BIP/GRP78 dissociation, PERK phosphorylates eukaryotic translation initiation factor 2α (eIF2α), which exerts an inhibitory effect on protein translation initiation (Harding et al., 1999). The reduction of protein synthesis affects the level of unfolded proteins, thus helping the ER folding machinery to act more efficiently. However, some proteins are preferentially translated and maintaining the synthesis of, among the others, ATF4, which activates key genes regulating protein folding, amino acid metabolism, redox control, and ERGIC-53, which regulates the ER-to-Golgi cargo protein trafficking (Blais et al., 2004; Spatuzza et al., 2004; Renna et al., 2006; Amodio et al., 2009).

BIP/GRP78 disassociation enables IRE1α activation, which retains both kinase and endoribonuclease activity, and the activation of its paralog IRE1β, for which PERK also induces translational repression by specifically controlling 28S rRNA turnover (Iwawaki et al., 2001). Instead, active IRE1α drives the mRNA splicing of a 26-nucleotide from X-box binding protein 1 (XBP1) (Calfon et al., 2002), which restores the translation of XBP1 that enhances the expression of UPR-dependent genes, including that for ER folding (calnexin, calreticulin), ERAD factors (*HERPUD1* [homocysteine inducible ER protein with ubiquitin-like domain 1], *EDEM1* [ER degradation-enhancing alpha-mannosidase-like protein 1]), autophagy components (*ATG5*, *ATG12*, *BECN1* [beclin 1], *UVRAG* [UV radiation resistance-associated]) and redox metabolism (*SOD1* [superoxide dismutase 1], catalase, *TRX1* [thioredoxin]) (Acosta-Alvear et al., 2007; Liu et al., 2009).

The third UPR component is ATF6 p90. Upon release from BIP/GRP78, ATF6 is directed to the *cis*-Golgi, where it is proteolytically cleaved. The active ATF6 fragment (ATF6 p50 or nATF6) activates the transcription of not only BIP/GRP78, GRP94/GP96, and XBP1, but also the cell death activator CHOP, and other proteins such as the calcium pump SERCA (sarcoplasmic/ER calcium ATPase pump) and p58IPK/DNAJC3 (Haze et al., 1999; van Huizen et al., 2003; Renna et al., 2007; Ron and Walter, 2007).

However, in prolonged or severe ER stress UPR may lead to apoptosis (Zhang and Kaufman, 2006; Walter and Ron, 2011). A primary determinant of apoptosis is PERK–eIF2α–ATF4 signaling (Rutkowski et al., 2006), which culminates in the induction of CHOP/GADD153 (Zinszner et al., 1998), which eventually lead to apoptotic cell death (Ohoka et al., 2005; Puthalakath et al., 2007).

Under acute stress, IRE1α is also involved in apoptosis and programmed cell death. Here, IRE1α activation induces JNK (c-Jun N-terminal kinase) activation through the formation of a tripartite complex from IRE1, ASK1, and TNF receptor-associated factor 2 (TRAF2) (Nishitoh et al., 2002).

Thus, UPR plays either a protective or a lethal role in response to proteotoxicity. Intriguingly, UPR pathways may regulate the expression of several genes related to neurodegenerative disease. For example, XBP1 activates the expression of AD-related genes such as those encoding for subunits of the γ-secretase complex or cyclin-dependent kinase 5 (CDK5) (Acosta-Alvear et al., 2007) and can influence amyloid precursor protein (APP) processing (Yang et al., 1998; Domingues et al., 2007). Interestingly, a more recent report proposed a model describing a vicious circle in which the activation of JNK3 by ER stress in AD increased amyloid-β production, amplifying in turn the ER stress response (Yoon et al., 2012). Furthermore, the UPR PERK–eIF2α axis can improve APP processing through β-secretase 1 (O’Connor et al., 2008), whereas ATF4 can regulate presenilin expression and γ-secretase activity (Mitsuda et al., 2007; Ohta et al., 2011).

In PD, ATF4 regulates Parkin expression (Bouman et al., 2011); Parkin itself controls the transcription of another PD-associated gene (*PARK7*, [parkin RBR E3 ubiquitin protein ligase]) through XBP1 (Duplan et al., 2013).

**FIGURE 2:**
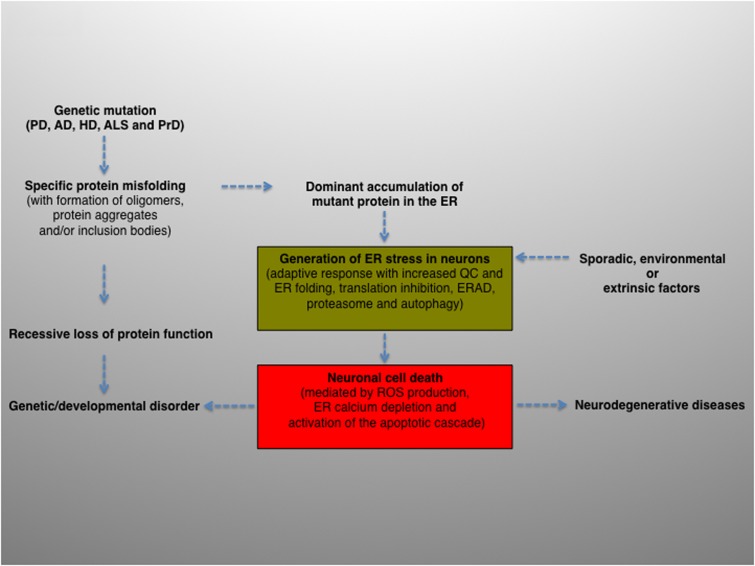
Schematic diagram depicting the potential role and contribution of ER stress in the pathogenesis of neurodegenerative diseases. ER activity is a major contributing factor to the overall biological fitness and homeostasis of eukaryotic cells. Consequently, ER stress and UPR are intimately interconnected with the pathogenesis of several human diseases and clinically relevant conditions. The activation of this pathway can derive from genetic or environmental factors, or both. For example, ER stress can result from a genetic mutation that causes a direct defect (i.e., folding, activity, post-translational modification, transport) of certain secretory proteins. More frequently, the progressive accumulation of misfolded and aggregation-prone mutant proteins with the formation of oligomers, aggregates and inclusion bodies represents a common feature of many age-related conditions, including neurodegenerative diseases. Neuronal protein aggregation can lead to recessive loss of function, which could contribute to the etiology of developmental and degenerative disorders. In this context, the accumulation of unfolded or misfolded proteins can affect the compartment at different levels, generating ER stress. The consequent activation of UPR will encompass an initial phase, characterized by an adaptive response aimed at alleviating the ER protein overload and preserving neuronal viability (green box), followed by a second, non-reversible response whereby the ER stress–mediated cell death program will ultimately be activated (red box), leading to neuronal loss. Finally, sporadic, extrinsic or environmental insults (e.g., physical injuries and neurotoxins) can perturb the ER, induce neuronal UPR and ultimately contribute to the pathogenesis of neurodegenerative diseases.

To summarize, intracellular protein aggregation is a salient feature underlying various neurological diseases. The balance between protein generation and degradation is crucial for protein homeostasis. Intriguingly, while most aggregated proteins result in the common endpoint of neuronal impairment, each aggregated protein has a distinct mechanism of action. Therefore, distinct molecular mechanisms may converge and contribute, albeit to a different extent, to ER proteostasis imbalance and ultimately cause neurodegeneration (**Figure 2**).

## Crosstalk between UPR and Autophagy in Neurodegenerative Diseases

Increasing evidence suggests that compromised autophagy contributes to the pathogenesis of various neurodegenerative diseases. As many neurodegenerative disease-associated aggregate-prone proteins are autophagy substrates, the levels of these proteins increase in both the soluble and aggregated state when autophagy is compromised (Rubinsztein et al., 2012; Menzies et al., 2015). Furthermore, mitochondrial damage activates an autophagy pathway named mitophagy and it is linked to neurodegenerative diseases such as PD and ALS (Valente et al., 2004; Chung et al., 2016).

In PD, heterozygous mutation of *GBA1* (glucocerebrosidase) is the most common known genetic risk factor. *GBA1* encodes GBA, a lysosomal enzyme that cleaves the β-glucosyl linkage of glucosylceramide (GlcCer). Loss of GBA activity with resultant increased GlcCer levels led to increased α-syn levels in cultured neurons. Increased α-syn in turn inhibited lysosomal maturation and GBA activity, resulting in additional GlcCer accumulation and further α-syn accumulation (Mazzulli et al., 2011). Interestingly, in addition to inhibiting the autophagy-lysosomal pathway, *GBA* mutations activate UPR and lead to ER stress in fibroblasts and iPSC-derived DA neurons from patients with PD (Fernandes et al., 2016; Sanchez-Martinez et al., 2016). In contrast, in ALS pathogenesis intracellular aggregates correlate with autophagosome accumulation and decreased proteasome activity (Cheroni et al., 2009; Chen et al., 2012).

A significant number of studies indicate that UPR is intimately linked to autophagy (Kouroku et al., 2007; Adolph et al., 2013). As the continuously adjusted homeostatic balance between protein folding/synthesis and degradation is of particular importance in neuronal cells, autophagy has become a relevant target for disease intervention in most neurodegenerative diseases (Wong and Cuervo, 2010; Green and Levine, 2014). Many studies have proven that UPR manipulation has clear consequences in substrate degradation by autophagy. For example, IRE1 regulates mutant huntingtin (HTT) clearance and most studies have shown that IRE1 can control autophagy levels by recruiting the adaptor protein TRAF2 and activating MAPK8 (mitogen-activated protein kinase 8)/JNK (Fouillet et al., 2012). Another report indicates that poly-Q trait expression triggers PERK activation and the phosphorylation of eIF2α, activating autophagy possibly due to the up-regulation of *ATG12*, an essential autophagy regulator (Kouroku et al., 2007). Moreover, ATF4 induces *ATG12*, *ATG5*, and *BECN1* expression, providing a direct link between UPR signaling and the control of autophagy (B’Chir et al., 2013).

Instead, XBP1 deficiency enhanced neuronal survival and the motor performance of the HD mice, with a mechanism of protection that does not appear strictly related to the ER stress response, but rather depending on up-regulated autophagy. Indeed, such regulation is independent of XBP1, but is due to the targeting of FOXO1 (forkhead box O1), a key transcription factor modulating autophagy in neurons that is regulated negatively by XBP1 *in vivo* (Zhou et al., 2011; Vidal et al., 2012; Zhao et al., 2013). Hence, these findings are indicative of critical crosstalk between the UPR IRE1–XBP1 arm and FOXO1–autophagy in HD neurons, providing a novel link between two major stress pathways and suggesting possible therapeutic benefits of targeting this pathway in a disease context.

Huntington disease is a late-onset neurodegenerative disease, characterized by specific mutations in the polyglutamine trait (poliQ expansion) contained in the first exon of the huntingtin protein. These mutations drastically change the hydrophobic properties of the protein, leading to the formation of large and insoluble inclusions within neurons (Arrasate and Finkbeiner, 2012). Even though toxic mHTT aggregates are primarily eliminated through autophagy, autophagy dysfunction is often observed in HD pathogenesis. In this context, the ectodermal-neural cortex 1 (ENC1) has been recently identified as a novel binding partner of p62/SQSTM1, which negatively regulates autophagy (Lee H. et al., 2016). Interestingly, under ER stress the expression of ENC1 and the ENC1/p62 interaction is enhanced by the IRE1/TRAF2/JNK pathway. Furthermore, ENC1 co-localizes with mHTT aggregates, thus inhibiting cargo recognition of p62 and leading to the accumulation and neurotoxicity of mtHTT aggregates. Accordingly, knockdown of ENC1 expression enhances co-localization of p62 with mtHTT aggregates, ameliorates the ER stress-induced impairment of autophagy and relieves neuronal cell death (Lee H. et al., 2016).

Growing evidence correlates defects in the autophagy system with ALS pathogenesis. ALS is predominantly sporadic, although a growing number of genes have been identified in the familial forms. ALS-associated mutations in *TDP43*, *SOD1*, *FUS*, and *C9ORF72* (C9 open reading frame 72) (Watabe and Nakaki, 2004; Andersen and Al-Chalabi, 2011; Fecto and Siddique, 2011; Farg et al., 2014) result in protein misfolding, aggregate accumulation and is associated with ER stress (Dafinca et al., 2016). Furthermore, these intracellular aggregates correlate with autophagosome accumulation and decreased proteasome activity in the neurons and brain tissues from patients with ALS (Cheroni et al., 2009; Chen et al., 2012). Alterations in *C9ORF72* are the most common cause of ALS and FTD and are linked by common pathological features (DeJesus-Hernandez et al., 2011). Wild-type *C9ORF72* is involved in regulating endocytic transport and colocalises with the autophagic proteins in human motor neurons (Farg et al., 2014), and interacts with the autophagy receptors p62/SQSTM1 (sequestosome 1) and OPTN (optineurin) and affects autophagosome formation (Sellier et al., 2016). In a recent report the kinase HIPK2 has been identified as the essential link that promotes ER-stress-induced cell death via the IRE1α-ASK1-JNK pathway. In SOD1 mutant mice, loss of HIPK2 delays disease onset, reduces cell death in spinal motor neurons and improves survival. Conversely, blocking HIPK2 kinase activity protects motor neurons from TDP-43 cytotoxicity, revealing a previously unrecognized role of HIPK2 activation in ER-stress-mediated neurodegeneration and its potential role as a therapeutic target for ALS (Lee S. et al., 2016).

Overall, the current experimental evidence sheds light on how two fundamental homeostatic processes, i.e., the UPR pathway and autophagy, contribute to cellular stress management and provide evidence in favor of therapeutic strategies for manipulating UPR levels, which may have broad beneficial consequences for alleviating neurodegeneration.

## Alteration of ER Proteostasis and Protein Trafficking as Possible Causes of Neurodegeneration

Global alteration in ER proteostasis can have an inhibitory effect on ER–Golgi trafficking (Renna et al., 2006; Amodio et al., 2009, 2013). As neuronal cells rely on the fitness of the secretory compartments for their function, impaired protein trafficking could also cause neurodegenerative disorders. Indeed, several findings suggest that ER retention of disease-related proteins can be decisive for neuronal cell death. This can occur in AD models (Katayama et al., 1999; Nakagawa et al., 2000) and in PD or familial CJD (Ugolino et al., 2011; Xu and Zhu, 2012).

In animal models and in the brain tissues from patients with PD (Colla et al., 2012a,b), mutant α-syn accumulates in the ER, where it is retained by BIP/GRP78 (Colla et al., 2012a) and can impair protein trafficking by affecting RAB1 (Ras-related protein Rab-1A) and SNAREs function at the ER–Golgi boundary (Cooper et al., 2006; Gitler et al., 2008; Thayanidhi et al., 2010). Similarly, mutant FUS and TDP43 interact with PDI (protein disulfide isomerase) both in experimental models and in tissues from patients with ALS (Farg et al., 2012; Walker et al., 2013).

In this context, envisaging that ER chaperones may act by reducing protein aggregation and therefore sustaining neuronal survival is conceivable (Atkin et al., 2006; Kikuchi et al., 2006; Walker et al., 2010).

Intriguingly, disease-related proteins that also accumulate in the cytosol, e.g., mutant SOD1 and HTT, can impair the ERAD machinery, induce ER stress and alter protein trafficking (Turner et al., 2005; Urushitani et al., 2006; Duennwald and Lindquist, 2008; Nishitoh et al., 2008; Yang et al., 2010; Hetz, 2012). A similar effect was reported for phosphorylated tau protein in AD models (Abisambra et al., 2013). Furthermore, cytosolic HTT accumulation can perturb different levels of the secretory pathway (del Toro et al., 2006; Vidal and Hetz, 2012). In addition, non-physiological protein–protein interactions can determine at different levels a number of distinct perturbations along the secretory pathway, generating chronic ER stress (**Table 1**). One example is the ALS-related mutant form of vesicle-associated protein B (VAPB) that accumulates and is retained within the ER, and the cargo receptor protein YIF1A (Yip1-interacting factor homolog A), which is normally localized along the secretory pathway in the ERGIC (Kuijpers et al., 2013). Moreover, an ALS-linked mutant VAPB variant affects the normal ER localization of quality control components (Moustaqim-Barrette et al., 2014). Finally, the expression of disease proteins such as VAPB and HTT can affect the three-dimensional organization of the ER tubular cisternae, ultimately contributing to the dysfunction of this compartment (Fasana et al., 2010). Furthermore, recent studies have identified mutations and a functional association between sigma non-opioid intracellular receptor 1 (SIGMAR1) and both FTD and ALS (Luty et al., 2010; Al-Saif et al., 2011). SIGMAR1 knockdown impairs ER–Golgi vesicular trafficking, ultimately leading to reduced autophagosome–lysosome fusion and reduced autophagy substrate degradation (Vollrath et al., 2014). Furthermore, it is worth mentioning how interplay between anterograde transport along the secretory pathway and the autophagosome/lysosome-dependent clearance of disease-causing proteins has also been reported in AD and HD experimental disease models (Lee et al., 2010; Renna et al., 2011).

**Table 1:** Disturbance of ER homeostasis as a conduit to neurodegenerative diseases.

ER process	Mutated protein	Genetic disease	Mechanism	Reference
Protein folding/activity	α-Synuclein (α-Syn)	PD	Interaction with BiP	Bellucci et al., 2011; Colla et al., 2012a,b
	Superoxide dismutase 1 (SOD1)	ALS	Interaction with BiP and PDI	Atkin et al., 2006
	Mutant fused in sarcoma (FUS); TDP-43	ALS	Interaction with PDI	Farg et al., 2012; Walker et al., 2013
	Glucocerebrosidase (GBA1)	PD	Reduced enzymatic activity	Mazzulli et al., 2011; Fernandes et al., 2016; Sanchez-Martinez et al., 2016
Glycosylation/retro-translocation/ERAD	Huntingtin (Htt)	HD	Interaction with ERAD components	Yang et al., 2010
	Superoxide dismutase 1 (SOD1)	ALS	Interaction with ERAD components	Nishitoh et al., 2008
	Phosphorylated tau	AD	Interaction with ERAD components	Abisambra et al., 2013
Vesicular trafficking	α-Synuclein (α-Syn)	PD	Interaction with RAB1 and inhibit the exit of vesicles from the ER	Cooper et al., 2006; Gitler et al., 2008
	ATP13A2	PD	Inhibits vesicular traffic between the ER and Golgi	Ugolino et al., 2011
	VAPB	ALS	Sequesters YIF1A and wild-type VAPB (VAPB^WT^), both of which are required for trafficking	Kuijpers et al., 2013
	Sigma non-opioid intracellular receptor 1 (SIGMAR1)	ALS, FTD	Inhibits vesicular traffic between the ER and Golgi; reduces lysosomal activity and autophagosome clearance	Luty et al., 2010; Al-Saif et al., 2011; Vollrath et al., 2014
UPR activation	XBP1	AD, bipolar disorder	A polymorphism on the XBP1 promoter reduces XBP1 transcription levels	Kakiuchi et al., 2003; Liu et al., 2013
	VAPB	ALS	Reduces the activation of inositol-requiring enzyme 1 (IRE1) and ATF6α	Kanekura et al., 2006; Gkogkas et al., 2008; Suzuki et al., 2009
	Presenilin-1 (PS1)	AD	Reduces the activation of inositol-requiring enzyme 1 (IRE1)	Katayama et al., 1999; Niwa et al., 1999
Calcium homeostasis	Huntingtin (Htt)	HD	Enhance calcium release possibly by interacting with IP3R; activation of RYR receptor	Tang et al., 2003; Higo et al., 2010;Chen et al., 2011
	α-Synuclein (α-Syn)	HD	Inhibition IP3R function	Belal et al., 2012; Selvaraj et al., 2012

Hence, and despite the fact that the mechanisms have not been fully delineated, it is clear how the fine regulation of membrane trafficking events are intimately connected with general proteostasis of the ER. Consequently, these alterations can affect the ER and ultimately contribute to neurodegeneration.

To summarize, both experimental and clinical evidence support the causative role of three UPR branches in the pathogenesis of neurodegenerative diseases (**Table 1**). As UPR activation and impaired synaptic plasticity occur across a spectrum of neurodegenerative disorders, it presents an attractive therapeutic target in these increasingly prevalent diseases. However, based on the nature of the disease, the sequence of events mediated by the aggregated proteins can differ greatly.

For example, in AD, Aβ is the primary source of ER stress, whereas in PD accumulated α-syn first induces cellular responses such as reactive oxygen species (ROS) generation and oxidative stress, which in turn mediate secondary ER stress, leading to disease pathogenesis. Similarly, these studies suggest how the three individual UPR modules can exert different effects, ranging from an adaptive or protective response to apoptosis, depending on the disease. A better understanding of the specific response in mediating aberrant protein aggregation is thus critical for dissecting the mechanism(s) underlying disease pathogenesis and for future development of therapeutic targets. Nevertheless, as outlined in the following sections, the three arms of the UPR pathway represent potential targets for treating neurodegenerative diseases.

## Impaired UPR Machinery as a Cause of Neurodegeneration

So far, few reports have provided evidence of a direct correlation between defective UPR effectors and neurodegeneration. Initial studies have suggested that presenilin-1 possibly inhibits IRE1 function (Katayama et al., 1999; Niwa et al., 1999), while *XBP1* promoter polymorphism has been indicated as an AD risk factor (Liu et al., 2013) and for the development of bipolar disorders and schizophrenia in a genetic association study (Kakiuchi et al., 2003). Consistent with this, a more recent study has further reinforced the evidence that the IRE1/XBP1 signaling axis can exacerbate AD pathogenesis (Duran-Aniotz et al., 2017).

Interestingly, the existence of a possible role played by ER stress and UPR modulators in the pathogenesis of clinically relevant conditions such as bipolar disorder and schizophrenia is further highlighted by two other genetic association studies, where mutations or SNPs have been identified in the genes encoding the ER-resident chaperones calreticulin and GRP94/GP96 (Aghajani et al., 2006; Kakiuchi et al., 2007). Also, *PDI* variants (Kwok et al., 2013), *UBQLN2* and p62/*SQSTM1* mutations participate in ALS development (Deng et al., 2011; Teyssou et al., 2013). Consistent with this, the ER-resident chaperone BIP/GRP78 has been recently shown to be able to reduce protease-resistant PrP (PrPSc) accumulation and propagation *in vitro* and *in vivo*, suggesting that modulating its levels/activity may offer a novel opportunity for designing therapeutic approaches for these diseases (Park et al., 2017).

Furthermore, disease-associated proteins can interact with integral UPR components thus interfering with the signaling system. For example, the ALS-linked VAPB variant interacts with and inhibits ATF6 transcriptional activity (Gkogkas et al., 2008) and IRE1- and XBP1-mediated signaling activity, and ultimately influences cell susceptibility to ER stress (Kanekura et al., 2006; Suzuki et al., 2009). Conversely, mutant HTT expression selectively inhibits ATF6 but not the other UPR transducers (Fernandez-Fernandez et al., 2011). Similarly, manipulation of the PD-associated *LRRK2* homolog in *Caenorhabditis elegans* led to altered BIP/GRP78 expression and high susceptibility to ER stress, while a mutant *LRRK2* induced ER stress-dependent apoptotic cell death (Samann et al., 2009; Yuan et al., 2011).

Collectively, speculating how strategies aimed at alleviating ER stress and restoring ER proteostasis may have much potential benefit on the outcomes of neurodegenerative diseases is conceivable. However, they also imply how the possible relations between UPR impairment and the pathogenesis of some neurodegenerative diseases are complex and not necessarily univocal. On one hand, the precise mechanisms still require delineation in many cases. In particular, identifying the optimal target(s) to reduce ER stress in each neurodegenerative disease is of fundamental importance. On the other hand, it is worth mentioning that different experimental models can also account for the apparently contradictory (or unexpected) effects reported, implying that a thorough analysis of the pathological state is necessary for predicting and minimizing any possible side effect deriving from experimental manipulation of the UPR.

## Impairment of ER Ca^2+^ Homeostasis and Er/Mitochondria Interplay in Neurodegenerative Diseases

Along with the mitochondria, the ER represents the major intracellular Ca^2+^ store. Many ER resident proteins require Ca^2+^ to preserve their function (Berridge et al., 2003; Krantic et al., 2005; Clapham, 2007). ER Ca^2+^ depletion leads to either down-regulation or overexpression of ER-resident factors (Amodio et al., 2011, 2016). Indeed, excessive Ca^2+^ efflux from ER stores has been reported in several pathologies of the central nervous system, including AD and PD (Mekahli et al., 2011; Rosenberg and Spitzer, 2011).

Despite the existence of different indirect means of inducing Ca^2+^ release from the ER via signaling events, direct ER Ca^2+^ channel perturbation has been associated with PMDs. For example, the non-physiological association between HTT and IP3R results in altered ER Ca^2+^ homeostasis (Tang et al., 2003; Higo et al., 2010). Similarly, RyR inhibition conferred significant neuroprotection in animal models of HD (Chen et al., 2011). In PD, α-syn over-expression impaired ER Ca^2+^ homeostasis and led to IP3R and RyR degradation (Belal et al., 2012). This observation agrees with the altered Ca^2+^ channel expression that has been described in both PD animal models and in tissues from patients with PD (Belal et al., 2012; Selvaraj et al., 2012). Finally, by sensitizing cells to ER stress, the over-expression of misfolded PrP drastically decreases ER Ca^2+^ availability (Torres et al., 2010).

Another important mechanism for regulating Ca^2+^ homeostasis is guaranteed by crosstalk between SERCA and store-operated Ca^2+^ entry (SOCE). In this context, the stromal interaction molecule proteins (STIMs) are fundamental SOCE regulators and are essential contributors to Ca^2+^ levels and homeostasis (Soboloff et al., 2012). Hence, and considering the high neuronal sensitivity to ER Ca^2+^ store perturbations, SOCE has been proposed as a potential target for gaining neuroprotection by reversing the ER stress-induced damage that often precedes neurodegeneration. Interestingly, a recent study has shown how STIM1 over-expression can reconstitute SOCE and confer protection against ER and oxidative stress induced by SERCA pharmacological blockade (Zhang and Thomas, 2016). These findings thus suggest that SOCE could indeed represent an additional mediator of neuroprotection, in particular under conditions where Ca^2+^ imbalance elicits ER stress (Zhang and Thomas, 2016).

Over previous years, ER and mitochondria contact sites have attracted much attention in the context of neurodegenerative disorders (McInnes, 2013). Neurons are critically dependent on mitochondria-associated membranes as a means of exchanging metabolites and signaling molecules between these organelles. Therefore, the processes affected by the ER and mitochondria contact sites are widely implicated in neurodegeneration, and an increasing number of disease-associated proteins have been reported to physically associate with the ER-mitochondria interface and cause structural and/or functional perturbations of this compartment (Krols et al., 2016; Erpapazoglou et al., 2017).

In AD Aβ affects both the ER and mitochondria and disrupts cellular Ca^2+^ homeostasis, and mitochondrial dysfunction is one of the main pathological events in AD. The mitochondria accumulate Aβ derived from the ER or Golgi compartment or from the ER–mitochondria contact sites, also known as mitochondria-associated ER-membranes (MAM) and synaptic mitochondria show very high vulnerability suggesting that the translocation of misfolded proteins to the mitochondrial membrane might play an important role in either triggering or perpetuating neurodegeneration (Schreiner et al., 2015).

In this context, MAM play a fundamental role in ER-mitochondria crosstalk, and mitochondria along with the UPR transducers and ER-localized Ca^2+^ receptors could represent a potential target for AD treatment (Volgyi et al., 2015; Penke et al., 2016).

## Targeting UPR as a Potential Therapeutic Tool in Neurodegenerative Disorders

The UPR pathway can represent a valuable therapeutic target for controlling the ER stress response associated with a growing group of clinically relevant conditions, including neurodegenerative diseases. In recent years, many chemical compounds and small molecules capable of alleviating or inducing ER stress through different mechanisms have been identified (Wang and Kaufman, 2014) (summarized in **Table 2**).

**Table 2:** ER stress and UPR pathways as therapeutic targets.

Small molecule or genetic approach	Molecular target	Potential or validated application	Reference
Azoramide	–	Alleviate ER stress by enhancing the folding capacity of the ER	Fu et al., 2015
Bip inducer X	Bip	Alleviate ER stress by enhancing the folding capacity of the ER	Kudo et al., 2008
GSK 2606414	PERK inhibitor	Abolishes translation attenuation and counteracts neurodegeneration in mouse AD model; neuroprotective in both pink1 and parkin PD models	Moreno et al., 2013;Celardo et al., 2016
ISRIB	Integrated stress response inhibitor, prevents PERK activation during ER stress	Increases cognitive function and ameliorate cognitive defects deriving from neurodegenerative diseases	Sidrauski et al., 2013, 2015; Halliday et al., 2015
Trazodone hydrochloride dibenzoylmethane	Integrated stress response inhibitor, prevents PERK activation during ER stress	Prevents neurodegeneration in mouse models of prion disease and a form of familial tauopathy (frontotemporal dementia – FTD)	Halliday et al., 2017
Sephin 1	PPP1R15A, which stimulates stress-induced eIF2α PP1 to dephosphorylate eIF2α	Prevents the motor, morphological, and molecular defects of Charcot-Marie-Tooth 1B and ALS	Das et al., 2015
Guanabenz	Interact with the protein phosphatase, Pp1/Gadd34 and blocks eIF2α dephosphorylation	Protective in fibroblasts expressing G93A mutant SOD1	Vieira et al., 2015
Salubrinal	Inhibition of PERK/elF2α-dependent ER stress	Suppress Aβ-induced neuro-inflammatory responses and ameliorates function in AD	Hong et al., 2016
Allicin	Increase the expression of PERK and its downstream effector Nrf2	Protective role in AD model	Zhu et al., 2015
Ambroxol/Isofagomine	Glucocerebrosidase	Improves phenotype of GBA1-associated PD models	Sanchez-Martinez et al., 2016
β -Asarone	Reduce UPR signaling	PD rat model	Ning et al., 2016
4-BPA/Ryanodine	RyR receptor	Reduced Ca^2+^-dependent ER stress and loss of dopaminergic neurons in PD model	Huang et al., 2017
XBP1 gene therapy	AAV-mediated delivery of active XBP1	Reduced the accumulation of mutant Htt in a mouse model of HD	Matus et al., 2009
PERK ablation	Reduce eIF2α phosphorylation	AD mouse model	Ma et al., 2013
Puma, Bip, ASK1 ablation	PERK downstream signaling	Protection against experimental ALS	Hetz et al., 2007;Nishitoh et al., 2008
XBP1 ablation	XBP1 downstream signaling	Protection in ALS and HD models	Hetz et al., 2009; Vidal et al., 2012
IRE1 RNAse domain ablation	IRE1 downstream signaling	Protection in AD model	Duran-Aniotz et al., 2017
CHOP ablation/XBP1 over-expression	AAV-mediated delivery of CHOP RNAi/active XBP1	Protects from degeneration of the optical nerve in mouse model of traumatic injury and glaucoma	Yang L. et al., 2016

Chemical chaperones such as the antidiabetic compound azoramide (Fu et al., 2015) and BIP/GRP78 inducer X (BiX) (Kudo et al., 2008) can reduce ER stress by improving its folding capability. ER stress might also be relieved *in vivo* by gene therapy-based approaches. This is the case, for example, of active *Xbp1*, which substantially reduced the accumulation of mutant HTT in a HD mouse model (Matus et al., 2009).

Evidence from several experiments has revealed that the PERK/eIF2α phosphorylation axis can be considered the main pathway in the occurrence of neurodegenerative diseases, in which there is either lost or increased PERK function. Loss of PERK function due to *PERK* gene mutation occurs in a rare neurodegenerative disorder known as Wolcott–Rallison syndrome; immunohistochemical analyses have shown that the PERK deficiency generates peculiar pathological changes whose features resemble those observed in neurodegenerative conditions (Bruch et al., 2015).

Instead, the PERK signaling pathway is hyperactive in the majority of PMDs. Notably, a recent study recovered memory impairment via brain-specific ablation of PERK expression in an *in vivo* model of AD (Ma et al., 2013).

General protein synthesis attenuation has similar effects on neuronal functions, also via other kinases that can induce eIF2α phosphorylation. For example, GCN2 (general control non-derepressible 2 serine/threonine kinase 2), PKR (protein kinase RNA-activated) and HRI (heme-regulated eIF2α kinase) can also phosphorylate eIF2α at serine51, activate the selective translation of the *BACE1* and *ATF4* mRNAs and generate memory and synaptic plasticity repression. Moreover, a recent investigation suggested that phosphorylation of eIF2α in AD is modulated by PKR, rather than PERK (Lourenco et al., 2013). In an AD mouse model, GCN2 deficiency caused BACE1 and ATF4 over-expression and consequently greater formation of senile plaques. Moreover, PERK activation was considerably increased in response to *GCN2* deletion, suggesting that eIF2α phosphorylation might induce senile plaque accumulation in AD brains by attenuating protein synthesis (Devi and Ohno, 2013).

Thus, PERK-specific inhibitors such as GSK2606414 abolished translation attenuation and counteract neurodegeneration in a mouse model of PrD-related neurodegeneration (Moreno et al., 2013). The high selectivity of GSK2606414 and its ability to cross the blood–brain barrier render such a molecule a promising therapeutic tool for neurodegenerative disorders. Therefore, they are becoming an attractive model to investigate the self-propagating properties of protein misfolding in neurodegenerative diseases, a common feature of AD, PD and ALS (Goedert et al., 2010; Soto, 2012).

Similarly, ISRIB (integrated stress response inhibitor) can prevent PERK activation under persistent ER stress (Sidrauski et al., 2013). ISRIB is a potent drug-like small molecule that retains the ability to inhibit eIF2α phosphorylation, and such an effect correlates well with increased cognitive function in rodents. Moreover, ISRIB inhibition of the PERK pathway significantly reduces ATF4 expression and consequently that of its target gene *CHOP*; a similar inhibitory effect of ISRIB has been shown on two other eIF2α kinases: GCN2 and HRI (Sidrauski et al., 2013). This observation is of particular importance as ATF4 is a repressor of memory and long-term synaptic plasticity. Moreover, recent studies have revealed that ISRIB activates eIF2β by stabilizing eIF2β-activated dimers, suggesting that by modulating eIF2β function, ISRIB is a promising instrument for ameliorating the cognitive defects that develop from neurodegenerative diseases (Sidrauski et al., 2015). ISRIB has also been proven effective in a mouse model of prion-related disease with reduced toxicity as compared to the PERK inhibitor GSK2606414 (Halliday et al., 2015). Consistent with this, a more recent study where a library of 1040 FDA-approved drugs was screened for novel disease modulators revealed how trazodone hydrochloride, a licensed antidepressant, and dibenzoylmethane, a compound currently under clinical trial as an anti-cancer drug, respectively, prevented emergence of the signs of brain cell damage in prion disease and restored memory in FTD mouse models (Halliday et al., 2017).

PPP1R15A is another eIF2α phosphorylation regulator that stimulates stress-induced eIF2α PP1 (serine/threonine protein phosphatase) to dephosphorylate eIF2α. Thus the inhibition of PPP1R15A, which prolongs eIF2α phosphorylation, could confer beneficial effects for treating PMD. Recently Sephin1, a small molecule derivative of the hypotensive drug guanabenz (GBZ) was synthesized. GBZ, a well-known α2 adrenergic receptor agonist, also interacts with PP1/GADD34, selectively blocking eIF2α dephosphorylation. Indeed, GBZ treatment protected fibroblasts bearing the *SOD1* G93A mutation subjected to the ER stress (Vieira et al., 2015). As GBZ retains high affinity for α adrenergic receptors, its inhibition of UPR signaling cannot be considered exclusive. Further pharmacological studies have paved the way for the development of additional lead compounds. GBZ can bind and inhibit the PPP1R15A/PP1c complex, which is regulated in response to stress eIF2α phosphatase activity. This has led to the identification of Sephin1, which inhibits the PP1 regulatory subunit *in vivo* by specifically binding the PPP1R15A subunit of the complex. Moreover, Sephin1 prevented the molecular and neurological defects in Charcot-Marie-Tooth 1B and ALS models (Das et al., 2015).

Recent works in AD mouse models have suggested that a specific glutamate receptor (mGluR5) isoform may operate as a molecular target for extracellular Aβ–regulated long-term depression (LTD), synaptic plasticity and Aβ synaptic toxicity. In particular, the mGluR-LTD inhibition observed in animal models of AD could be antagonized by suppressing the PERK pathway. These data reinforce the concept that reversing the inhibition of protein synthesis mediated by the PERK/eIF2α axis may alleviate some cognitive deficits observed in, at least some, neurodegenerative conditions (Yang W. et al., 2016).

The PERK/eIF2α-dependent UPR branch is also involved in the Aβ-induced neuroinflammatory responses in astrocytes. In the same context, different compounds suppressed Aβ-induced neuroinflammation. For example, in Aβ-induced astrocytes, salubrinal inhibition of PERK/eIF2α-dependent ER stress and exposure to the neurosteroid progesterone (PG) significantly suppressed neuroinflammation by down-regulating PERK/eIF2α signaling (Hong et al., 2016). Natural compounds can also have protective activity in AD models. For example, allicin, a natural garlic extract, had a protective effect in a rat model of AD, as demonstrated by its ability to moderately increase the expression levels of both PERK and its downstream substrate NFR2 (Zhu et al., 2015).

By contrast, activation of the IRE1 UPR branch appears to benefit AD pathogenesis, as shown in an AD model of *Drosophila melanogaster*, in which *Xbp1* over-expression prevented Aβ toxicity (Casas-Tinto et al., 2011), as in a similar study in *C. elegans*, indicating that XBP1 is involved in defense against Aβ toxicity, presumably by increased autophagy (Safra et al., 2013). However, a recent study has shown a positive correlation between IRE1/XBP1 signaling axis activity and AD pathogenesis (Duran-Aniotz et al., 2017). Contrary to the initial expectations that IRE1 signaling may protect against AD, genetic ablation of the RNAse domain of *IRE1* in the nervous system significantly reduced APP expression and amyloid deposition, Aβ oligomer content and astrocyte activation. At molecular level, inhibiting IRE1 downstream signaling reduces the APP steady-state levels via its retention in the ER, followed by proteasome-mediated degradation.

Collectively, these findings reveal a so-far unanticipated role of IRE1 in AD pathogenesis, providing a novel therapeutic intervention target (Duran-Aniotz et al., 2017). Furthermore, inhibition of the IRE1/XBP1-mediated branch of ER stress by the natural compound β-asarone improved the phenotype in a rat model of PD (Ning et al., 2016).

In PD, PERK/eIF2α pathway activation represents the main cause of dopaminergic neuron loss in patients although there is little evidence of UPR activation from postmortem studies of patients with PD (Scheper and Hoozemans, 2015). Despite this, a recent work using a Parkinson-like disease mouse model has reported how sustained gene transfer–mediated ATF4 up-regulation in the dopaminergic neurons of the substantia nigra resulted in severe and caspase 3/7-dependent nigrostriatal degeneration, confirming that the PERK UPR branch plays an essential role in PD pathogenesis by activating dopaminergic neuronal loss (Gully et al., 2016). On the other hand, it is worth mentioning how genetic *ATF6* deletion potentiates the susceptibility to PD-inducing neurotoxins in PD experimental mouse models (Egawa et al., 2011; Hashida et al., 2012), while *Bip*/*Grp78* over-expression exerted neuroprotective effects (Gorbatyuk et al., 2012).

In PINK1- and Parkin-associated PD models, mitofusins cause enhanced ER stress signaling, acting as a molecular bridge interconnecting mitochondria damaged by mutant PINK1 and Parkin proteins to the ER membranes. PERK signaling suppression has a neuroprotective effect by reducing mitofusin contact with the ER. Furthermore, either pharmacological (the GSK2606414 PERK inhibitor) or genetic inhibition of the PERK pathway was beneficial in these experimental models of PD (Celardo et al., 2016).

Furthermore, a potential role for the RYR receptor in the dopaminergic neurons loss in the context of PD pathogenesis has been proposed in a recent study (Huang et al., 2017). In particular, the detrimental increase in cytoplasmic Ca^2+^ levels induced by 6-hydroxydopamine (6-OHDA) (a toxin capable to generate, *in vitro* and *in vivo*, some features of PD-associated neurodegeneration) can be antagonized by pretreatment with the ER stress inhibitor 4-PBA or ryanodine, which acts as a blocker on the eponymous receptor (RYR). Notably, the pretreatment with 4-PBA and ryanodine proved to be effective also in DA neurons derived from midbrain sections of 6-OHDA-treated rat, therefore suggesting suggest a potential therapeutic strategy for PD by inhibiting the RyRs Ca^2+^ channels on the ER (Huang et al., 2017).

Concerning more specifically the familial form of PD associated with *GBA* gene mutations, a promising study has revealed the efficacy of two small molecules with chaperone activity, namely ambroxol and isofagomine (Sanchez-Martinez et al., 2016). In particular, both chaperones increased GBA levels and activity in fly models and in fibroblasts from patients with PD. Treatment with both chaperones reduced ER stress and prevented motor function loss, proof of principle that small molecule chaperones can reverse mutant *GBA*-mediated ER stress *in vivo* and might prove effective for treating PD (Sanchez-Martinez et al., 2016).

Remarkably, in ALS, genetic UPR manipulation and pharmacological approaches have revealed complex involvement of the pathways, illuminating distinct outputs of specific UPR signaling branches in the same disease (Matus et al., 2011). Importantly, in ALS mouse models, chronic ER stress represents one of the earliest pathological events in motor neurons before the appearance of ALS symptoms (Saxena et al., 2009).

According to the experimental evidence collected so far, targeting PERK signaling exerts a dual role in ALS. Indeed, eIF2α phosphorylation has a protective effect (Saxena et al., 2009; Wang et al., 2011), whereas ATF4 over-expression and the consequential up-regulation of the proapoptotic factors CHOP and BIM has definite detrimental effects (Matus et al., 2013). Targeting XBP1 protects cells against ALS by up-regulating autophagy, which controls the elimination of mutant SOD1 aggregates (Hetz et al., 2009). Similar results were reported in an XBP1 knockout mouse model of HD, in which up-regulated FOXO1, an important transcription factor controlling the early-stage activation of several autophagy genes, was observed (Vidal et al., 2012).

Another example of PERK/eIF2α involvement is the prion-related disorders (PrDs), in which prion infection increases eIF2α phosphorylation, which in turn reduces the expression of relevant synaptic proteins (Moreno et al., 2012). More interestingly, in animal models, a PERK inhibitor counteracted the pathophysiological role of eIF2α phosphorylation, protecting animals against PrD-caused neurodegeneration (Moreno et al., 2013). By contrast, either XBP1 or caspase-12 depletion did not affect disease progression and pathophysiology in models of PrD infection (Steele et al., 2007; Hetz et al., 2008). Of further note, a recent work has shown how the AAV-mediated combinatorial modulation of CHOP and active XBP1s has proved beneficial in rescuing the neurodegeneration of the optical nerve associated to both traumatic injury and glaucoma, thus expanding the potential application of UPR modulation to other classes of neurodegenerative conditions (Yang L. et al., 2016).

Altogether, these findings reinforce the idea of the complexity of UPR and how this pathway can affect certain diseases, which are largely influenced by flexible and versatile overlap between the selective UPR signaling branches, their downstream modules, and a number of genetic and/or environmental co-contributing factors. Moreover, they illustrate and strongly inform the need for thorough and accurate evaluation of the contribution of each individual UPR signaling component in PMDs, which will aid the definition of optimal targets for therapeutic intervention.

## Concluding Remarks and Future Directions

The identification of pharmacological therapies for neurodegenerative diseases remains an ambitious challenge in biomedical science and will become even more pressing in the future in view of the increased life expectancy of the aging population. The connections between ER stress, UPR and the pathogenesis of neurodegenerative disorders have been extensively studied in various disease models. These functional studies reinforce the concept that UPR can selectively modulate specific cellular events and this can in turn affect PMD progression through distinct mechanisms. Despite gaining numerous insights into the adaptive mechanisms for counterbalancing protein misfolding both at cellular and physiological level, many interesting aspects still have not been elucidated.

In this context, the role of UPR in the flexible control of ER proteostasis could be harnessed to find strategies aimed at attenuating or delaying the onset of neurodegenerative diseases. Indeed, prolonged albeit mild ER stress can induce an adaptive response, termed ER hormesis, which entails an UPR-dependent increase in ER proteostasis (Matus et al., 2012; Mollereau, 2013). However, additional studies have shown that this cellular response also involves autophagy, which decreases protein aggregates and cell death (Cullinan et al., 2003; Fouillet et al., 2012). For example, low dosages of tunicamycin attenuated neuronal degeneration by inducing the IRE1–XBP1 UPR axis (Fouillet et al., 2012). Similarly, neuronal ablation of *Xbp1* delayed in an autophagy-dependent manner, disease progression associated with SOD1 or mutant HTT (Matus et al., 2009; Vidal et al., 2012). Thus, it is conceivable that enhancing the adaptive mechanisms by slight induction of specific UPR pathways may offer sufficient protection against neurotoxicity.

Collectively, these studies strongly reinforce the concept that strategies aimed at attenuating ER stress levels may impact neurodegenerative diseases positively. As such, better understanding of the pathological state for each specific clinical condition is critical if the aim is to target UPR for treating neurodegeneration. Therefore, further studies will be needed to: (a) identify the optimal targets for attenuating the ER stress response to each specific clinical condition; (b) develop novel drugs; (c) predict and define possible side effects derived from UPR perturbations.

In this regard, both novel molecules and repurposed compounds have in the past few years been proven effective in different models of neurodegenerative diseases (Hetz et al., 2013). Although these findings are all of great interest *per se*, the identification of additional mechanisms capable of driving and modulating UPR signaling in health and disease will shed further light on the regulation of such a fundamental biological response and will hopefully lead to the identification of novel therapeutic targets.

## Author Contributions

MR and PR conceived the work, prepared the figures, wrote and edited the manuscript.

## Conflict of Interest Statement

The authors declare that the research was conducted in the absence of any commercial or financial relationships that could be construed as a potential conflict of interest.
